# Harnessing biocompatible chemistry for developing improved and novel microbial cell factories

**DOI:** 10.1111/1751-7915.13472

**Published:** 2019-08-06

**Authors:** Jian‐Ming Liu, Christian Solem, Peter Ruhdal Jensen

**Affiliations:** ^1^ National Food Institute Technical University of Denmark DK‐2800 Kgs. Lyngby Denmark

## Abstract

White biotechnology relies on the sophisticated chemical machinery inside living cells for producing a broad range of useful compounds in a sustainable and environmentally friendly way. However, despite the impressive repertoire of compounds that can be generated using white biotechnology, this approach cannot currently fully replace traditional chemical production, often relying on petroleum as a raw material. One challenge is the limited number of chemical transformations taking place in living organisms. Biocompatible chemistry, that is non‐enzymatic chemical reactions taking place under mild conditions compatible with living organisms, could provide a solution. Biocompatible chemistry is not a novel invention, and has since long been used by living organisms. Examples include Fenton chemistry, used by microorganisms for degrading plant materials, and manganese or ketoacids dependent chemistry used for detoxifying reactive oxygen species. However, harnessing biocompatible chemistry for expanding the chemical repertoire of living cells is a relatively novel approach within white biotechnology, and it could potentially be used for producing valuable compounds which living organisms otherwise are not able to generate. In this mini review, we discuss such applications of biocompatible chemistry, and clarify the potential that lies in using biocompatible chemistry in conjunction with metabolically engineered cell factories for cheap substrate utilization, improved cell physiology, efficient pathway construction and novel chemicals production.

## Graphical abstract

The combination of biocompatible chemistry, i.e. non‐enzymatic chemical reactions, with microbial metabolism represents an attractive and novel approach in the construction of novel microbial cell factories.

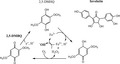

## Introduction

The massive development within the field of metabolic engineering, that has taken place over just a few decades, has allowed for the engineering of microorganisms that can efficiently synthesize a plethora of naturally and non‐naturally occurring chemicals (Stephanopoulos *et al*., 2004; Steen *et al*., 2010; Lee *et al*., 2012; Lee and Kim, 2015). Some of these microbial cell factories have already been applied in commercial‐scale production (Nakamura and Whited, 2003; Becker *et al*., 2011; Yim *et al*., 2011; Chen and Nielsen, 2013; Paddon *et al*., 2013; Van Dien, 2013). Despite the impressive chemical repertoire displayed by microorganisms, it is still relatively small when compared with what can be achieved using organic chemistry (Schwartz *et al*., 2014). Organic chemists have, over the last two centuries, developed a vast number of reagents, catalysts and reactions to facilitate synthesis of valuable molecules, most of which are not of natural origin.

Biological chemistry (enzymatic reactions) and organic chemistry (non‐enzymatic reactions) undoubtedly have their specific strengths and weaknesses. Bio‐based production normally offers environmentally friendly and sustainable biotechnological routes to chemicals, characterized by mild fermentation conditions, low‐cost and renewable feedstocks, high yields and excellent stereoselectivity (McDaniel and Weiss, 2005; Keasling and Abraham Mendoza, 2012). Gradually, it has become easier to manipulate the metabolism of an increasing number of microorganisms due to rapid development of genome engineering tools (Kim *et al*., 2016). However, there are also notable disadvantages associated with biological production, for example it is difficult to synthesize toxic or non‐naturally occurring compounds, or compounds where information regarding their biosynthesis is lacking. It is also a time‐consuming effort to develop some specific strains and economical processes that are really fit for commercial production (Hong and Nielsen, 2012; Davison and Lievense, 2016). But we are sure that the developing time would be significantly shortened with the development of revolutionary technologies in this field, such as systems metabolic engineering (Lee and Kim, 2015), robotic automation (Si *et al*., 2017) and the incorporation of artificial intelligence into synthetic biology (Nesbeth *et al*., 2016). In contrast, organic synthesis allows for comparatively rapid generation of a broad range of compounds, especially, some molecules derived from existing ones through chemical modification (Keasling and Abraham Mendoza, 2012). There are, however, often downsides to using this approach, such as harsh reaction conditions, use of toxic reagents and high costs for catalysts and for purifying the target compound. Is it possible to harness the best from both worlds? There are actually many examples that demonstrate this possibility, for example where traditional chemistry is used for modifying compounds of biological origin to produce pharmaceuticals (Lin *et al*., 2013; Paddon *et al*., 2013; a), biofuels (Anbarasan *et al*., 2012) and biomaterials (Suastegui *et al*., 2016), and there are comprehensive reviews describing these applications (Schwartz *et al*., 2014; Jambunathan and Zhang, 2015). The attractive technology to apply artificial metalloenzymes has also been recently and elegantly reviewed by Jeschek *et al*. (Jeschek *et al*., 2018). In this mini review, we focus on the use of biocompatible chemistry, that is mild chemistry compatible with the conditions of microbial life, for developing novel and improved microbial cell factories. By harnessing biocompatible chemistry, the repertoire of chemical reactions, which living organisms are capable of carrying out, can be greatly extended (Wallace and Balskus, 2014). Below, we provide examples of natural‐occurring as well as artificially designed applications of biocompatible chemistry. We specifically and briefly explain how biocompatible chemistry can be harnessed to extend metabolic capabilities of microorganisms at different levels: (i) substrate level: expanding the scope for cheap substrate utilization, like lignin and CO_2_; (ii) cell physiology level: promoting microbial growth under harsh conditions; (iii) product level: constructing novel metabolic pathways for valuable compounds. In addition to benefit bacteria, having biocompatible reactions in extremophiles that can tolerate high pressure, temperature or solvents could be envisioned as another interesting aspect in this area. The widespread occurrence of biocompatible chemistry paves the way for its various biotechnological applications.

## Expanding the scope for cheap substrate utilization

### Lignin

**Figure 1:**
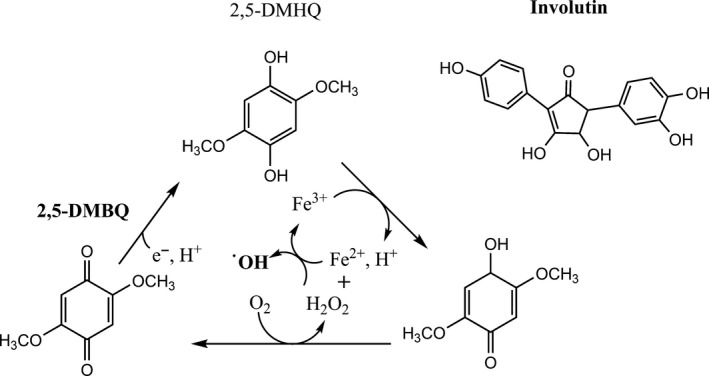
Harnessing biocompatible chemistry to promote growth. 2,5 dimethoxy‐1,4‐benzoquinone (2,5‐DMBQ) and involution are secreted by certain fungi, and stimulate Fenton chemistry which results in formation of highly reactive hydroxyl radicals (^·^OH) that can be used to degrade lignin.

Lots of efforts have been focused on engineering various microorganisms to convert abundant and renewable feedstocks into biofuels, biochemicals and bioenergy (Lee and Kim, 2015), as the cost of the feedstock, to a large extent, determines the economic feasibility of the biological process. Lignocellulose is one of the most abundant renewable feedstocks available. However, there are great challenges associated with the use of this particular feedstock, due to its recalcitrant nature. In order for microorganisms to make use of lignocellulose derived sugars, lignin, a complex aromatic polymer, first needs to be degraded. Some microbes have developed sophisticated approaches involving non‐enzymatic chemistry to promote lignin degradation. A good example is the brown rot fungus *Gloeophyllum trabeum*, which secretes the compounds 2,5‐dimethoxy‐1,4‐benzoquinone (2,5‐DMBQ) and 4,5‐DMBQ, that drive a Fenton reaction system generating highly reactive hydroxyl radicals (^·^OH) (Jensen *et al*., 2001) (Fig. 1). These hydroxyl radicals can easily penetrate wood polymers, oxidize lignin and thereby facilitate the degradation process. By harnessing the similar strategy, the ectomycorrhizal fungus *Paxillus involutus* secrets a different compound – involutin (Fig. 1), to degrade both polysaccharides and lignin as well (Shah *et al*., 2015). This type of biocompatible chemistry in these fungi offers a competitive advantage over other microorganisms with which they compete for nutrients.

### CO_2_

The direct CO_2_ fixation to form organic compounds is one renewable and sustainable solution for solving the global challenges, such as reducing the dependence on the fossil‐based industry and alleviating the climate warming problems (Liao *et al*., 2016). Biological systems, including plants, algae and microorganisms, always rely on the photosynthetic machinery to capture CO_2_ through the Calvin‐Benson‐Bassham (CBB) cycle (Chen *et al*., 2018). Although this system has been evolved over billions of years, many enzymes including Rubisco are exhibiting very low efficiencies (Li and Liao, 2013).

**Figure 2:**
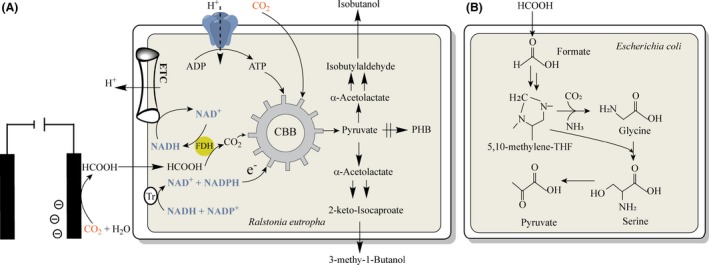
The integration of an electrochemical reaction with cellular metabolism for CO_2_ fixation. A. Formate is generated electrochemically from CO_2_ and H_2_O in the presence of Li catalyst. Formate is subsequently converted to CO_2_ and NADH by formate dehydrogenase (FDH) using an engineered *Ralstonia eutropha* strain. NADH is used for generating ATP via oxidative phosphorylation and NADPH through transhydrogenase, two compounds that are used to drive CO_2_ fixation to produce longer chain alcohols. B. The reductive glycine pathway. CBB, calvin‐benson‐bassham; FDH, formate dehydrogenase; PHB, polyhydroxybutyrate; THF, tetrahydrofolate; Tr, transhydrogenase.

To overcome these limitations, Li *et al*. (2012) elegantly designed a system, where the typical light reactions were decoupled from dark reactions, and successfully integrated an electrochemical reaction to mimic the light reactions providing the necessary driving forces (ATP and NADPH) for the dark reactions (Fig. 2A). The integrated process included electrochemical formation of formate from CO_2_, formate utilization by the engineered bacteria – *Ralstonia eutropha* for producing isobutanol and 3‐methy‐1‐butanol. By using only electricity and CO_2_ in one bioreactor, it was demonstrated that 140 mg l^−1^ isobutanol and 3‐methy‐1‐butanol were achieved (Liao and Li, 2013). This case clearly demonstrates the great potential that lies in using biocompatible chemistry, which here refers to electrochemical conversion of CO_2_ to formate, for capturing CO_2_ as well as the conversion of electricity, which currently cannot be stored efficiently, to generate valuable biofuels and biochemicals. One challenge associated with this approach is lacking of efficient formate metabolizing pathways in microbes. Bar‐Even summarized the naturally occurring and synthetic pathways that support formate assimilation (Bar‐Even, 2016). And one naturally occurring metabolic route, called reductive glycine pathway (Fig. 2B), has been recently constructed in engineered *Escherichia coli* that can convert formate into glycine and serine (Yishai *et al*., 2018). It can even support slight growth using only formate and CO_2_ (Bang and Lee, 2018). All these pioneering works open up novel possibilities to integrate CO_2_‐converted formate into the cheap feedstock for microbial fermentation.

## Promoting microbial growth under harsh conditions

### Oxidative stress

Oxidative stress is one of the most common forms of stress that microorganisms are exposed to, and for this reason the microbes have developed various enzymatic and non‐enzymatic defense systems against it. Enzymes such as catalase, superoxide dismutase (SOD) and peroxidases can eliminate some of the reactive oxygen species (ROS) generated, and these enzymes rely on different metal ions for their catalytic function (Aguirre and Culotta, 2012). Mn^2+^ (manganese) is required for some types of SOD, but surprisingly Mn^2+^ can function as a SOD non‐enzymatically without a protein partner by interacting with simple molecules. Archibald and Fridovich found that a Mn^2+^‐dependent ${\text{O}}_{2}^{-}$ scavenging activity in *Lactobacillus plantarum*, an organism which is devoid of an SOD, could be attributed to Mn complexes with phosphate and lactate (Archibald and Fridovich, 1981, 1982a,1982b). Furthermore, it was found that in SOD mutant strains of *Escherichia coli* and *Bacillus subtilis*, oxidative damage could be alleviated when cells were grown in Mn^2+^‐rich medium (Inaoka *et al*., 1999; Al‐Maghrebi *et al*., 2002). A yeast mutant lacking the cytosolic Cu/Zn‐dependent SOD1 enzyme, accumulated superoxide, and this had a negative effect on amino acid biosynthesis and increased the mutation frequency (Lapinskas *et al*., 1996). Also in this case growing the mutant in the presence of high Mn^2+^ concentrations had a beneficial effect (Chang and Kosman, 1989; Sanchez *et al*., 2005). When the intracellular Mn^2+^ concentration was below a certain level, aerobic growth was hampered (Reddi *et al*., 2009; Reddi and Culotta, 2011). It seems that this simple Mn^2+^‐dependent non‐enzymatic reaction is universal, and acts as a supplement or as a backup of SOD enzymes.

**Figure 3:**
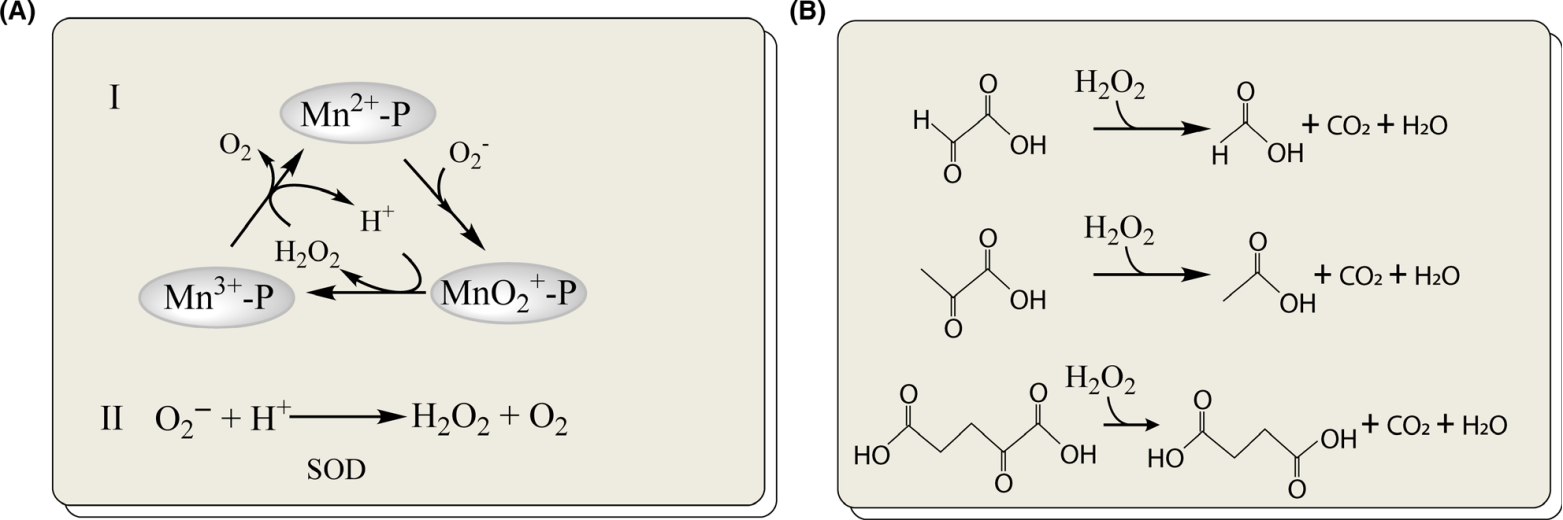
Proposed mechanism for Mn^2+^‐dependent and ketoacid‐dependent detoxification of ROS and. A. A Mn (II) complex, such as Mn^2+^‐P (phosphate or polyphosphate), can be oxidized by ${\text{O}}_{2}^{-}$ to a Mn (III) complex (Mn^3+^‐P) with the generation of H_2_O_2_, whereafter ${\text{O}}_{2}^{-}$ reduces Mn (III) to Mn (II) resulting in formation of O_2_. The superoxide dismutase (SOD) enzymatically can perform a similar detoxification of ROS. B. The ketoacids, such as glyoxylate, pyruvate and α‐ketoglutarate can non‐enzymatically be converted into corresponding organic acids formate, acetate and succinate in the presence of H_2_O_2_.

The exact chemical mechanism by which Mn^2+^ can detoxify ROS still remains to be elucidated in detail. *In vitro* experimental studies by Barnese *et al*. confirmed that Manganese phosphate and Manganese carbonate under physiologically relevant conditions can catalyse superoxide disproportionation (Barnese *et al*., 2012). For instance, the complex – Mn^2+^‐P (phosphate or polyphosphate) can be oxidized by ${\text{O}}_{2}^{-}$ to form Mn (III) – Mn^3+^‐P, while the formed H_2_O_2_ can reduce the Mn (III), as shown in Fig. 3A. *In vivo* studies carried out by McNaughton *et al*. showed that Mn^2+^ is bound to phosphates (Mn‐Pi) and the intracellular level of Mn‐Pi was associated with the ability to handle ROS (McNaughton *et al*., 2010). Mn‐Pi and several Mn porphyrins have been shown to be able to substitute for SOD1 in yeast (Munroe *et al*., 2007). The mechanism of how the microorganisms physiologically form the Mn complexes is unknown. In the past, it was assumed that cellular regulation was not involved in their formation. However, the study carried out by Reddi and Culotta (2011) demonstrated that, in *S. cerevisiae*, the nutrient‐sensing and stress response pathways tightly regulate the formation of Mn complexes. Specifically, the serine/threonine protein kinase, Rim 15, can sense and respond to nutrient signals, including the changes in phosphate levels via the interaction with Pho80/Pho85 kinase and nitrogen levels via the Sch9 kinase. Rim 15 then acts on transcription via the two transcription factors Gis1 and Msn2/4. The loss of Gis1 can result in enhanced formation of Mn‐P complexes, while the loss of Msn2/4 has a depressing effect.

In addition to Mn^2+^‐dependent non‐enzymatic antioxidation, it is reported that ketoacids are non‐enzymatically involved in ROS elimination. There are three main types of ketoacids that are pyruvate, α‐ketoglutarate and glyoxylate, which are intracellular metabolites and are able to perform non‐enzymatic decarboxylation in the presence of oxidizing agents (Fig. 3B) (Lemire *et al*., 2017). The soil microbe *Pseudomonas fluorescens* was able to increase the generation of pyruvate, α‐ketoglutarate and glyoxylate through metabolic reprogramming when exposed to ROS (Alhasawi *et al*., 2015). It was also found that the growth of low‐temperature‐stressed *E. coli* was significantly recovered when supplemented with peroxide degrading compounds like pyruvate or α‐ketoglutarate or catalase (Mizunoe *et al*., 1999). These observations point out that these ketoacids maybe commonly involved in combating oxidative stress in different microorganisms through biocompatible reactions. Harnessing these ketoacids as ROS scavengers could offer the organisms many benefits: (i) rapid adaptation to oxidative stress by adjusting the pools of these small metabolites. (ii) intracellular as well as extracellular ROS elimination, because these ketoacids and ROS can be secreted into the extracellular environment. (iii) recycling of the products, such as acetate produced from pyruvate oxidation and succinate produced from α‐ketoglutarate oxidation, readily into microbial metabolism.

### Electron transfer under anaerobic conditions

Promoting extracellular chemistry by secreting redox‐active compounds appears to be a common approach that many microorganisms rely on. It is well‐known that some facultative anaerobic microorganisms use extracellular compounds as terminal electron acceptors under oxygen‐limiting conditions, and in some cases, secreted compounds are involved in this. For instance, it has been found that *Shewanella algae* BrY secretes melanin, which serves as a soluble electron shuttle, to reduce insoluble iron oxide (Turick *et al*., 2002) and that *Pseudomonas chlororaphis* secretes redox‐active pigments, such as phenazines, which are also involved in electron‐transfer reactions (Pham *et al*., 2008). These extracellular electron‐transfer reactions facilitate anaerobic respiration and help support growth under these conditions.

Actually due to the ability to secrete such electron shuttles, many bacteria have found applications in microbial fuel cells (Rabaey and Verstraete, 2005). There is one common group of molecules known as flavins serving as excellent electron shuttles. For example, *S. oneidensis* produced riboflavin and when riboflavin was removed from biofilms, the electron‐transfer rate was reduced by more than 70% (Marsili *et al*., 2008). *P. aeruginosa* was able to secrete another type of flavin called pyocyanin and the concentration of pyocyanin had a direct correlation to power generation efficiency (Shen *et al*., 2014). It would be expected that the electron‐transfer efficiency could be improved by engineering these bacteria to produce more electron shuttles.

### Rescue of growth by synthesizing essential metabolites

Non‐enzymatic chemistry has also been used for rescuing auxotrophic microorganisms. For instance, *p*‐aminobenzoic acid (PABA) and *p*‐hydroxybenzoic acid (PHBA) are the essential precursors for the synthesis of folate and ubiquinone in microorganisms, respectively. Lee *et al*. (2013) developed two biocompatible reactions that directly can take place in the fermentation medium, either based on a ruthenium or an iron catalyst, and that can facilitate conversion of different precursors into essential metabolites PABA or PHBA. These catalysts enabled growth of *E. coli* mutants that were unable to synthesize these compounds. The biocompatibility of these organic or inorganic catalysts with microorganisms is yet another demonstration of the feasibility of incorporating biocompatible chemistry for promoting cellular growth and microbial survival.

## Producing valuable chemicals

### Food ingredients – Diacetyl and (*S*,* S*)‐2,3‐Butanediol

As mentioned, biocompatible chemistry is a useful add‐on to the chemistry of the living cell. We recently took advantage of this and demonstrated how it could be used, in conjunction with a metabolically engineered *Lactococcus lactis* strain, to produce the three industrially important chemicals diacetyl, (3*S*)‐acetoin and (*S*,*S*)‐2,3‐Butanediol (S‐BDO) (Liu *et al*., 2016a,2016b). *L. lactis* is a widely used dairy bacterium, with great potential to serve as an efficient microbial cell factory (Kandasamy *et al*., 2016; Liu *et al*., 2016c,2016d, 2017, 2019).

**Figure 4:**
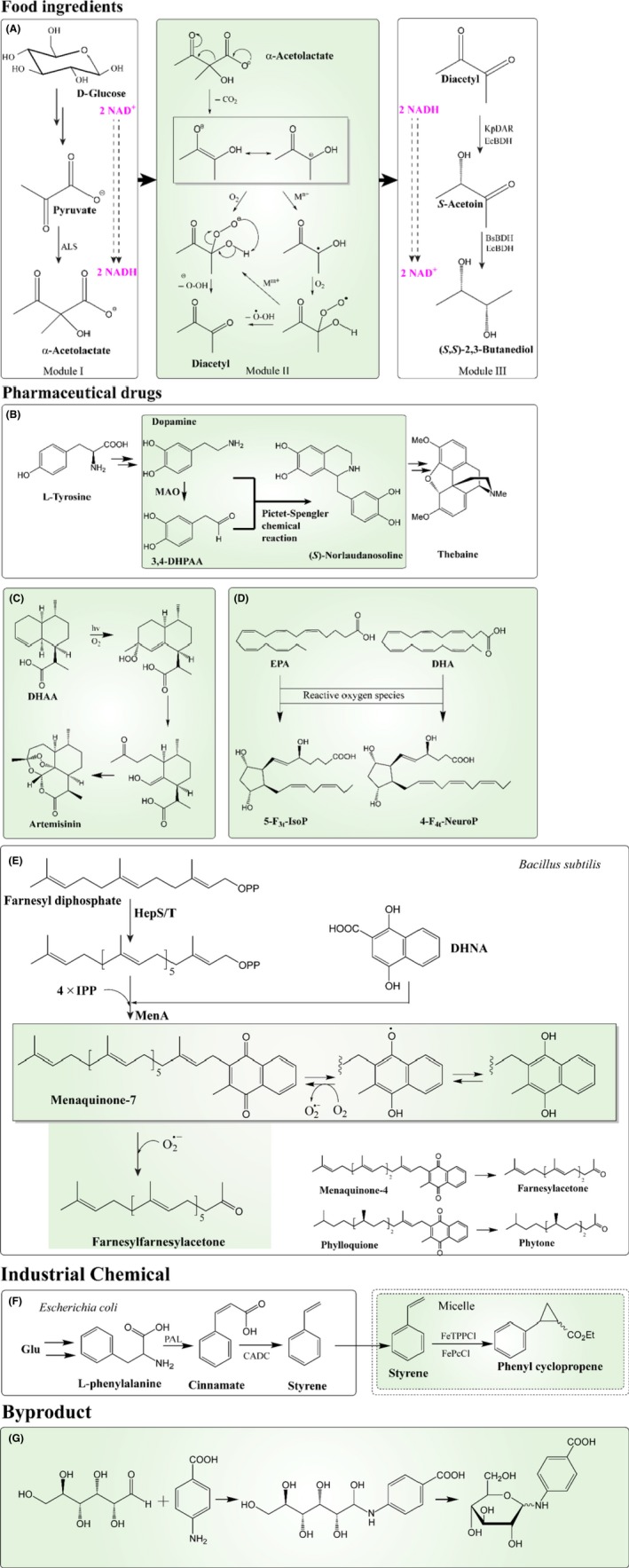
Producing unique chemicals by harnessing biocompatible chemistry. The biocompatible reactions involved are highlighted by green background. A. The non‐enzymatic oxidative decarboxylation from α‐acetolactate to diacetyl (Module II) links the two metabolic pathways (Module I: from glucose to α‐acetolactate by glycolysis and α‐acetolactate synthase; Module III: from diacetyl to *S*‐acetoin and further to (*S*,*S*)‐2,3‐butanediol by butanediol dehydrogenase). The synthetic pathway for (*S*,*S*)‐2,3‐butanediol is redox neutral. B. The Pictet–Spengler chemical reaction is involved in the condensation of dopamine and 3,4‐DHPAA (3,4‐dihydroxyphenylacetaldehyde) to form (*S*)‐norlaudanosoline, which is an essential precursor for the synthesis of thebaine. C. The non‐enzymatic chemical reactions are responsible for the final steps in the synthesis of artemisinin from dihydroartemisinic acid (DHAA). D. Non‐enzymatic oxygenated metabolites in the presence of reactive oxygen species, 5‐F_3t_‐isoprostanes (IsoP) from EPA and F_4t_‐neuroprostanes (NeuroP) from DHA. E. The production of norisoprenoid by combining enzymatic and non‐enzymatic reactions in *Bacillus* cells. Menaquinone‐7, which is synthesized by combination of heptaprenyl diphosphate and 1,4‐dihydroxy‐2‐naphtoate (DHNA) through enzymatic reactions, can react with superoxide non‐enzymatically to form farnesylfarnesylacetone, one non‐C5‐units terpenoid. Another two norisoprenoids farnesylacetone and phytone can be formed similarly from menaquinone‐4 and phylloquinone, respectively. F. In the presence of phthalocyanine catalyst, phenyl cyclopropane can be achieved from styrene, which is biosynthesized in metabolically engineered *E. coli*. The vitamin E‐derived micelles provide compartmentalized space to accommodate this chemical reaction to alleviate the toxicity of styrene and further increase the production of phenyl cyclopropane with high titre and productivity. G. Non‐enzymatic glycosylation between an aldehyde group in glucose and an amino group in *para*‐aminobenzoate (PABA). It was found as a byproduct for producing PABA in engineered *C. glutamicum*.

Diacetyl is a food ingredient with strong buttery aroma, while (3*S*)‐acetoin and S‐BDO are valuable stereoisomers that have various applications (Lee *et al*., 2011; Xiao and Lu, 2014). However, the fermentative production of these compounds has not been fully explored, especially for the latter two, where no previously described fermentative approaches have been reported. For diacetyl, one of the challenges is its slow formation from α‐acetolactate via non‐enzymatic oxidative decarboxylation. The conversion of α‐acetolactate to diacetyl can, however, be accelerated by various metal catalysts (Fe^3+^, Fe^2+^ or Cu^2+^) (Mohr *et al*., 1997). We combined metabolic engineering of *L. lactis*, which included the inactivation of all the competitive pathways by deleting the genes encoding lactate dehydrogenase (LDH), phosphotransacetylase (PTA), alcohol dehydrogenase (ADHE), α‐acetolactate decarboxylase (ALDB) and diacetyl reductase (DAR)/butanediol dehydrogenase (ButBA), with biocompatible chemistry by adding metal catalysts to achieve the high‐titre and high‐yield production of diacetyl. Subsequently, we extended the metabolic pathway from diacetyl to (3*S*)‐acetoin and S‐BDO by the introducing DAR/BDH from *Enterobacter cloacae*. In this way, biocompatible chemistry was used to link two metabolic pathways (Module I & III, Fig. 4A), by accelerating conversion of α‐acetolactate to diacetyl (Module II), thereby allowing for synthesis of S‐BDO, in a redox neutral fashion and with high yield.

### Potential pharmaceuticals

In this part, various metabolic pathways in combination with biocompatible chemistry will be further illustrated for producing bioactive compounds and potential pharmaceutical drugs. It includes (i) amino acid‐derived pathway for thebaine synthesis from tyrosine, (ii) IPP (isopentenyl pyrophosphate)‐derived terpenoid pathway with artemisinin as an example, (iii) Non‐C5‐units terpenoid pathway for synthesis of norisprenoid, (iv) fatty acid‐derived pathway for cyclic‐oxygenated metabolites.

Thebaine is the precursor for opioid production and opioid has many medical applications for pain relief as well as palliative care (Thodey *et al*., 2014). The biosynthesis of thebaine is derived from amino acid – tyrosine, which undergoes enzymatic reactions to form dopamine and 3,4‐dihydroxyphenylacetaldehyde (3,4‐DHPAA). The non‐enzymatic Pictet–Spengler reaction is able to combine dopamine with 3,4‐DHPAA to produce (*S*)‐norlaudanosoline (Fig. 4B), which is an essential precursor for thebaine (Nakagawa *et al*., 2015). The Pictet–Spengler reaction, therefore, links the upstream and downstream metabolic pathways for the complete formation of thebaine. In addition to the non‐enzymatic reaction, the norcoclaurine synthase from *Thalictrum flavum* was discovered to be able to catalyse the Pictet–Spengler reaction (Lichman *et al*., 2017), which provides another possibility for its complete biosynthesis.

In diverse terpenoid families, artemisinin is one of the most effective therapeutic drugs and is used as a standard treatment for malaria. Great efforts have been used to produce this compound through a completely biosynthetic pathway, but presently the microbial production is terminated at artemisinic acid (AA), which requires the subsequent chemical conversion to artemisinin (Ro *et al*., 2006; Paddon *et al*., 2013). It was discovered non‐enzymatic chemical reactions are responsible for the final steps in the synthesis of artemisinin (Fig. 4C), in particular from dihydroartemisinic acid (DHAA) (Sy and Brown, 2002; Czechowski *et al*., 2016). So harnessing and accelerating these non‐enzymatic reactions in microbes could provide a complete pathway for artemisinin.

Recently, a non‐C_5_‐unit terpenoid, farnesylfarnesylacetone (C33 norisoprenoid), was proposed to be synthesized by combining enzymatic and non‐enzymatic chemistry (Ueda *et al*., 2018). Norisoprenoids are widely distributed in nature as insect hormones and pheromones, plant odorants and microbial volatiles. Some of them are potential antiulcer drugs (Hirakawa *et al*., 1996). In *B. megaterium* and *B. subtilis*, Ueda *et al*. proposed that superoxide mediated the cleavage of menaquinone‐7 in a non‐enzymatic way to synthesize farnesylfarnesylacetone (Fig. 4E). Another two norisoprenoids farnesylacetone and phytone can be formed similarly from menaquinone‐4 and phylloquinone, respectively.

Docosahexaenoic acid (DHA) and eicosapentaenoic acid (EPA) are long chain polyunsaturated fatty acids and they have beneficial effects on human health. Metabolic engineering of *Yarrowia lipolytica* has been reported to produce DHA and EPA (Xue *et al*., 2013; Zhu and Jackson, 2015). Due to the abundance of double bonds in the structure of DHA and EPA, they can undergo non‐enzymatic peroxidation to produce cyclic‐oxygenated metabolites under oxidative stress conditions (Roy *et al*., 2016). These metabolites, such as 5‐F3‐isoprostanes (IsoP) from EPA and F4‐neuroprostanes (NeuroP) from DHA (Fig. 4D), are potential bioactive drugs for treating neurological disorders such as Alzheimer's disease and Parkinson's disease (Becker, 2004).

### Important industrial chemical – cyclopropane

It was reported that a biocompatible cyclopropanation reaction was used for generating the non‐natural phenyl cyclopropane directly from styrene in the presence of an iron phthalocyanine catalyst (FeTPPCl or FePcCl) (Wallace and Balskus, 2015). The styrene was produced using a metabolically engineered *E. coli* strain (a phenylalanine overproducer), in which phenylalanine ammonia lyase (PAL) from *Arabidopsis thaliana* and trans‐cinnamic acid decarboxylase (CADC) from *Saccharomyces cerevisiae* had been introduced to redirect the flux from phenylalanine to styrene (McKenna and Nielsen, 2011). More interestingly, they modified this fermentation system (including the catalysts) by adding vitamin *E*‐derived designer micelles (Fig. 4F), which provided compartmentalized space for biocompatible chemistry to convert styrene to phenyl cyclopropene (Wallace and Balskus, 2016). This elegant design alleviates the toxicity of styrene, enables the production of phenyl cyclopropene from glucose directly with higher titre and productivity and provides an example on how to avoid toxic effects from catalysts or reactions on microorganisms. This case illustrates that biocompatible chemistry can be used to expand the variety of chemicals that can be derived from renewable and sustainable feedstocks, particularly those of industrial importance that are not easily made using conventional enzyme‐catalysed chemistry.

### Byproducts

As mentioned above, non‐enzymatic chemistry can be used to complement and expand the synthesis capability of microbial cell factories for producing valuable products, it is also possible to synthesize non‐targeted byproducts. Kubota *et al*. found that when they engineered *Corynebacterium glutamicum* for the production of PABA, an N‐glucosyl byproduct was formed spontaneously, which was caused by an non‐enzymatic chemistry between an amino group in PABA and an aldehyde group of glucose (Fig. 4G) (Kubota *et al*., 2016). Because this glycation reaction was reversible, the byproduct can be converted to PABA when glucose concentration was low or treated by acid. Although *N*‐glucosyl PABA is considered as a side product compared with PABA, having this non‐enzymatic reaction can benefit the cells to produce higher titres of PABA, because *N*‐glucosyl PABA is much less toxic than pure PABA and the glycosylated form can be easily converted to the desired product. This byproduct‐formation case actually offers advantages for producing more target products. Another example also lies in the synthesis of PABA from chorismate. Chorismate is also the precursor for aromatic amino acid production. Winter *et al*. (2014) knocked out the genes *ARO7/PHA2* responsible for the conversion from chorismate to phenylpyruvate in order to drive the chorismate flux into PABA, but they found that phenylpyruvate can be formed non‐enzymatically by chorismate rearrangement. Therefore, understanding these non‐enzymatic reactions could help us design rational approaches to increase the production of target chemicals.

## Extremophiles with biocompatible chemistry

As a large number of chemical reactions (non‐enzymatic) require high temperature, high pressure or extremely acidic (or alkaline) conditions, these requirements are not compatible with the growth conditions for most of the bacteria. However, there are some extremophiles isolated from extreme environments that can tolerate these conditions with high biocompatibility. So harnessing these non‐enzymatic chemical reactions in extremophiles would be promising for expanding their surviving or synthesis capacities. For example, in seafloor hydrothermal vents where the temperature is extremely high and the hydrothermal fluid is composed of minerals (Fe, Zn, Mn and Cu) and small molecules (H_2_, H_2_S, CH_4_), the inorganic oxidation/reduction reactions play key roles for producing simple organic compounds, such as formate, amino acid and pyruvate (Zierenberg *et al*., 2000). It is reported several hyperthermophilic archaea belonging to the *Thermococcus* genus, that are isolated from deep‐sea hydrothermal field, are capable of oxidizing formate to produce ATP for surviving with the coproduction of H_2_ (Kim *et al*., 2010). The diversity of life in these extreme environments provides clues for life evolution and also represents an opportunity to exploring extremophiles in combination with biocompatible chemistry for biotechnological applications (Di Donato *et al*., 2018).

## The widespread non‐enzymatic chemistry

Although the interaction between non‐enzyme‐catalysed chemistry and cellular metabolism has caught little attention, it is in fact a ubiquitous and frequent event. Keller *et al*. summarized that the non‐enzymatic reactions could occur in all the six major enzymatic classes: oxidoreductases, transferases, hydrolases, lyases, isomerases and ligases (Keller *et al*., 2015). The representative examples, which are present in the central metabolic pathways, include the non‐enzymatic isomerization between glucose‐6‐phosphate and fructose‐6‐phosphate in the glycolysis pathway (Gracy and Noltmann, 1968), the non‐enzymatic hydrolysis of 6‐phosphogluconolactone to 6‐phosphogluconate in the pentose phosphate pathway (PPP) (Rakitzis and Papandreou, 1998). Most of these non‐enzymatic reactions are spontaneous or accelerated by small metal catalysts, and can occur in parallel with enzymatic pathways. There are also specific non‐enzymatic reactions, during which biocompatible chemistry occurs as the only pathway. A well‐known example is the synthesis of vitamin D3, where the precursor 7‐dehydrocholesterol is converted to pre‐vitamin D3 by UV‐light‐induced isomerization followed by spontaneous isomerization to mature vitamin D3 (Deluca, 2014). The widespread non‐enzymatic reactions have been proposed to play important roles in the origin of life (Keller *et al*., 2014). These non‐enzymatic reactions are also particularly relevant to cellular metabolism under stress conditions, for example ferrous iron‐catalysed conversion of hydrogen peroxide to hydroxyl radical under oxidative stress (Valko *et al*., 2005), non‐specific protein modifications and amino acid conjugations under heat stress or exposure to UV (Keller *et al*., 2015), protein lysines acylated with reactive thioesters (e.g. acetyl‐CoA) that accumulates and becomes ‘carbon stress’ as defined by Wagner and Hirschey (2014). Although many non‐enzymatic reactions have been substituted by enzymatic counterparts in the process of evolution, it is quite interesting to investigate the opportunities to intentionally use biocompatible chemistry for extending metabolic capabilities of microorganisms.

## Conclusions and future perspectives

The incorporation of biocompatible chemistry into microbial metabolism provides a novel approach to construct various microbial cell factories. These biocompatible non‐enzymatic reactions could facilitate the degradation of recalcitrant lignin through Fenton chemistry, the detoxification of ROS by Mn‐dependent chemistry and also expand the repertoire of compounds that can be produced by pure enzymatic transformation or pure organic synthesis as well as drive CO_2_ fixation in a comparably efficient manner. The widespread role of biocompatible chemistry in cellular metabolism paves the way for its various biotechnological applications.

To transform the knowledge in biocompatible chemistry into a transferrable technology that is systematically applicable to diverse scenarios and biological systems, the critical next step would be to construct databases compatible with the state‐of‐the‐art systems biology and develop computational methods for identifying novel pathways that exploit biocompatible chemistry. For example, all chemical reactions that can convert compounds with structural similarity to any metabolites present in cells under relatively mild conditions (within the biological realm) can be collected. Advanced atom mapping algorithms, for example, canonical labelling for clique approximation (Kumar and Maranas, 2014), minimum‐weighted edit‐distance (Latendresse *et al*., 2012), are able to analyse the mechanisms of reactions at the atomic level. A database for reactions with atom mappings and compounds with potential biocompatibility can be constructed. From the reaction atom mappings and chemical structures of compounds, novel reactions on unforeseen metabolites can be predicted using powerful computational techniques (e.g. machine learning) that have been increasingly employed to predict enzyme promiscuity (e.g. de Groot *et al*., 2009; Jeffryes *et al*., 2015; Mu *et al*., 2011). With this information integrated into the metabolic networks of production organisms, novel pathways combining metabolic reactions and non‐enzymatic reactions can be explored. Computational frameworks similar to OptStrain (Pharkya *et al*., 2004), OptForce (Chowdhury *et al*., 2014), etc. that take into account of the metabolic network structure as well as the microbial growth can also be developed specifically for biocompatible chemistry. These *in silico* tools along with wet‐laboratory experiments will be able to reveal the impact of non‐enzymatic reactions on cellular metabolism at the system level and inspire innovative strategies for combining metabolic engineering and biocompatible chemistry.

## Conflict of interests

None declared.
